# Protocol of a prospective multicenter study on comorbidity impact on multiple sclerosis and antibody-mediated diseases of the central nervous system (COMMIT)

**DOI:** 10.3389/fimmu.2024.1380025

**Published:** 2024-07-02

**Authors:** Sara Samadzadeh, Rafl Adnan, Paulina Berglova, Mahdi Barzegar, Birgit Debrabant, Stine Gundtoft Roikjaer, Michael Levy, Axel Petzold, Jacqueline Palace, Eoin P. Flanagan, Sara Mariotto, Soeren T. Skou, Anne Froelich, Itay Lotan, Silvia Messina, Ruth Geraldes, Susanna Asseyer, Hadas Stiebel-Kalish, Frederike Cosima Oertel, Vahid Shaygannejad, Mohammad Ali Sahraian, Ho Jin Kim, Jeffrey L. Bennett, Chotima Böttcher, Hanna G. Zimmermann, Brian G. Weinshenker, Friedemann Paul, Nasrin Asgari

**Affiliations:** ^1^ Institute of Regional Health Research and, Institute of Molecular Medicine, University of Southern Denmark, Odense, Denmark; ^2^ The Center for Neurological Research, Department of Neurology Slagelse Hospital, Slagelse, Denmark; ^3^ Charité – Universitätsmedizin Berlin, Corporate Member of Freie Universität Berlin and Humboldt-Universität zu Berlin, Experimental and Clinical Research Center, Berlin, Germany; ^4^ Neurosciences Research Center, Isfahan University of Medical Sciences, Isfahan, Iran; ^5^ Department of Mathematics and Computer Science, Faculty of Natural Sciences, University of Southern Denmark, Odense, Denmark; ^6^ The Research and Implementation Unit PROgrez, Department of Physiotherapy and Occupational Therapy, Næstved-Slagelse-Ringsted Hospitals, Slagelse, Region Zealand, Denmark; ^7^ Department of Neurology, Massachusetts General Hospital and Harvard Medical School, Boston, MA, United States; ^8^ The National Hospital for Neurology and Neurosurgery, and Moorfields Eye Hospital and Queen Square University College London (UCL), Institute of Neurology, London, United Kingdom; ^9^ Department of Neurology, Amsterdam The University Medical Center Utrecht (UMC), Amsterdam, Netherlands; ^10^ Department of Ophthalmology, Amsterdam The University Medical Center Utrecht (UMC), Amsterdam, Netherlands; ^11^ Nuffield Department of Clinical Neurosciences, Oxford University Hospitals, National Health Service Trust, Oxford, United Kingdom; ^12^ Department Neurology and Center for Multiple Sclerosis (MS), and Autoimmune Neurology, Mayo Clinic, Rochester, MN, United States; ^13^ Neurology Unit, Department of Neurosciences, Biomedicine, and Movement Sciences, University of Verona, Verona, Italy; ^14^ Center for Muscle and Joint Health, Department of Sports Science and Clinical Biomechanics, University of Southern Denmark, Odense, Denmark; ^15^ Innovation and Research Centre for Multimorbidity, Slagelse Hospital, Slagelse, Denmark; ^16^ Section of General Practice, Faculty of Health and Medical Sciences, University of Copenhagen, Copenhagen, Denmark; ^17^ Department of Neurology and Neuroimmunology Unit, Rabin Medical Center, Petah Tikva, Israel; ^18^ Tel Aviv University Faculty of Medicine, Tel Aviv University, Tel Aviv, Israel; ^19^ Experimental and Clinical Research Center, a cooperation between the Max Delbrück Center for Molecular Medicine in the Helmholtz Association and Charité Universitätsmedizin Berlin, Berlin, Germany; ^20^ Max Delbrück Center for Molecular Medicine in the Helmholtz Association (MDC), Berlin, Germany; ^21^ NeuroCure Clinical Research Center, Charité – Universitätsmedizin Berlin, Corporate Member of Freie Universität Berlin and Humboldt-Universität zu Berlin, Berlin, Germany; ^22^ Department of Ophthalmology, Neuro-Ophthalmology Unit, Rabin Medical Center, Petah Tikva, Israel; ^23^ Eye Laboratory, Felsenstein Research Institute, Tel Aviv, Israel; ^24^ Multiple Sclerosis (MS) Research Center, Neuroscience Institute, Tehran University of Medical Sciences, Tehran, Iran; ^25^ Department of Neurology, Research Institute and Hospital of National Cancer Center, Goyang, Republic of Korea; ^26^ Department of Neurology and Ophthalmology, Programs in Neuroscience and Immunology, University of Colorado Anschutz Medical Campus, Aurora, CO, United States; ^27^ Einstein Center Digital Future, Berlin, Germany; ^28^ Department of Neurology, University of Virginia, Charlottesville, VA, United States; ^29^ Open Patient data Explorative Network, Odense University Hospital, University of Southern Denmark, Odense, Denmark

**Keywords:** multiple sclerosis, neuromyelitis optica spectrum disorder, myelin oligodendrocyte glycoprotein antibody-associated disease, antibody-mediated diseases of the central nervous system, comorbidity

## Abstract

Comorbidities in patients with multiple sclerosis (MS) and antibody-mediated diseases of the central nervous system (CNS) including neuromyelitis optica spectrum disorder (NMOSD), and myelin oligodendrocyte glycoprotein (MOG)-antibody-associated disease (MOGAD) are common and may influence the course of their neurological disease. Comorbidity may contribute to neuronal injury and therefore limit recovery from attacks, accelerate disease progression, and increase disability. This study aims to explore the impact of comorbidity, particularly vascular comorbidity, and related risk factors on clinical and paraclinical parameters of MS, NMOSD and MOGAD. We propose COMMIT, a prospective multicenter study with longitudinal follow-up of patients with MS, NMOSD, and MOGAD, with or without comorbidities, as well as healthy subjects as controls. Subjects will be stratified by age, sex and ethnicity. In consecutive samples we will analyze levels of inflammation and neurodegeneration markers in both fluid and cellular compartments of the peripheral blood and cerebrospinal fluid (CSF) using multiple state-of-the-art technologies, including untargeted proteomics and targeted ultrasensitive ELISA assays and quantitative reverse transcription polymerase chain reaction (RT-qPCR) as well as high-dimensional single-cell technologies i.e., mass cytometry and single-cell RNA sequencing. Algorithm-based data analyses will be used to unravel the relationship between these markers, optical coherence tomography (OCT) and magnetic resonance imaging (MRI), and clinical outcomes including frequency and severity of relapses, long-term disability, and quality of life. The goal is to evaluate the impact of comorbidities on MS, NMOSD, and MOGAD which may lead to development of treatment approaches to improve outcomes of inflammatory demyelinating diseases of the CNS.

## 1 Introduction

Inflammatory demyelinating diseases (IDDs) of the central nervous system (CNS) are immune-mediated disorders that lead to the destruction of myelin, either primarily [multiple sclerosis (MS) and myelin oligodendrocyte glycoprotein-antibody-associated disease (MOGAD)] or secondarily [neuromyelitis optica spectrum disorder (NMOSD)]. There is a variable degree of axonal injury and loss, which is the ultimate substrate of disability (1, 2). The development and pathogenesis of IDDs are complicated immune mechanisms, presumably involving both innate and adaptive immunity, reflecting defects in autoimmune tolerance mechanisms. Likewise, the immunobiology of injury and repair is multifactorial and influenced by genetic and environmental factors. Remyelination may protect against axonal degeneration by oligodendrocyte precursor cell (OPC) proliferation and maturation to oligodendrocytes (OLs) in the case of actual OL loss to a variable degree (3). OLs have high metabolic requirements to maintain myelin synthesis and metabolic support of axons (3). The most well-defined IDDs are multiple sclerosis (MS), widely believed to be T-cell-mediated, and NMOSD and MOGAD, both of which are believed to be antibody-mediated.

Clusters of chronic conditions and comorbidities may occur in IDDs and are an area of current interest (4–12). Comorbidity refers to the burden caused by the presence and severity of any distinct additional entity other than the specific index disease and may result from similar predisposing genetic or environmental factors (13) or from treatment of IDDs (14, 15). For instance, corticosteroids can precipitate the onset of diabetes and hyperglycemia, while other certain immunosuppressive drugs are linked to an increased risk of cardiovascular disease (4). The co-occurrence of two or more chronic conditions, i.e., so-called multimorbidity, may be present even at the time of diagnosis (4).

In IDDs, comorbidity may contribute to remyelination failure and neuronal injury and adversely affect a broad range of outcomes, including the risk of relapse, diminished quality of life (QoL), and long-term disability.

Multimorbidity is a growing concern, with an increase in the prevalence of chronic somatic conditions as well as mental disorders, in part reflecting an aging population (10–12, 16, 17). Many factors such as lifestyle and socioeconomic status may lead to the onset of multimorbidity at a younger age (13). Emerging evidence underscores a high prevalence of comorbidity, i.e., vascular comorbidities and related risk factors in patients with multiple sclerosis (18), exemplified by type 2 diabetes mellitus (T2DM), which is a highly prevalent disorder worldwide. Patients with T2DM are at high risk for microvascular complications, owing to hyperglycemia and insulin resistance (metabolic syndrome) (19). Oral antidiabetic drugs such as metformin and pioglitazone may beneficially impact MS disease progression by reducing inflammatory mediators such as interferon-γ, interleukin (IL)-6, and TNF-alpha (20). T2DM may influence brain function and may cause brain insulin resistance, neuroinflammation including microglia activation, blood–brain barrier (BBB) impairment, and mitochondrial dysfunction, resulting in neuronal metabolic defects and neuronal cell death (21). The most common comorbidities in patients with NMOSD are hypertension and cardiovascular disease (4, 22), although uniquely in NMOSD, other autoimmune conditions, such as systemic lupus erythematosus, rheumatoid arthritis, Sjögren’s syndrome, and autoimmune encephalitis, may occur as concurrent comorbid diseases because of defective B-cell development checkpoints (5, 6). MOGAD has only recently been recognized and may be regarded as a relatively rare disorder (23), so the pattern of comorbidity has not yet been elucidated; however, anti-N-methyl-D-aspartate receptor encephalitis (anti-NMDAR encephalitis) and autoimmune thyroid disorders have been reported to occur (7).

Investigations of the impact of comorbidities on clinical outcomes of IDD have only been conducted to a limited extent. Comorbid conditions can be associated with a prolonged interval between the disease onset and its subsequent diagnosis. An increase in the frequency of relapses has been observed in NMOSD patients with comorbidities compared to NMOSD patients without comorbidities, with severe motor episodes and severe optic neuritis (ON) (15). Multifocal CNS lesions as a presenting symptom are more prevalent in patients with comorbidities compared to those without. Moreover, comorbidity can be indicative of reduced treatment responsiveness, heightened disability, and an elevated mortality rate (15, 24).

However, the current knowledge is mainly based on retrospective and cross-sectional studies with a small number of patients. A prospective and longitudinal study with an evaluation of the interaction between clinical and paraclinical parameters of comorbidity of subjects with MS, NMOSD, and MOGAD will be a timely effort.

### 1.1 Advancing data collection and protocols for comorbidity analysis and management

The high occurrence of comorbidities suggests screening for concomitant medical conditions as a routine part of patient care, as comorbidities may influence the clinical features, prognosis, and treatment outcomes. So far unexplored, the comorbidity and certain clusters of chronic conditions may have a negative impact on the levels of proinflammatory and neurodegenerative mediators, and they may negatively influence disease progression. Such knowledge will be important for optimizing treatment strategies and thereby hopefully improve QoL. Comorbidities may vary depending on age, sex, ethnicity, geographic region, socioeconomic composition, and healthcare system, highlighting the importance of a multicenter design. Furthermore, analytical pipelines for the determination of molecular and cellular markers as well as downstream algorithm-based (integrative) data analysis have been established and are in the process of validation. This multicentric study will analyze data from different sets of patients without or with one or more comorbidities, with follow-up visits. We aim to determine the levels of inflammatory and neurodegenerative mediators in patients with MS, NMOSD, and MOGAD, both with and without comorbidities, and evaluate whether changes may affect disease outcomes. Furthermore, we aim to assess whether comorbidity influences optical coherence tomography (OCT) and magnetic resonance imaging (MRI) parameters and its association with clinical outcomes.

## 2 Methods and analysis

### 2.1 Study design

The study comprises a prospective, multicenter investigation of patients with MS, AQP4-IgG-positive NMOSD, and MOGAD, with or without comorbidities with 3 years of follow-up in collaboration with multiple centers from Denmark (2), Germany (2), the USA (4), UK (1), Iran (2), Italy (1), Israel (1), and South Korea (1). All patients will be offered follow-up after 6, 12, 18, 24, and 36 months after baseline. The baseline is defined as the time of entry into the study, i.e., any time point during the disease course.

The COMMIT study will adhere to the STROBE (Strengthening the Reporting of Observational Studies in Epidemiology) guidelines for reporting (25). Approval will be sought from local ethical institutional review boards in all centers, and informed consent will be obtained from the patients. The study will be conducted in accordance with the provisions of the Declaration of Helsinki and the guidelines for good clinical practice set forth by the International Conference on Harmonization (26).

#### 2.1.1 Study population and diagnosis

We will recruit MS (27), NMOSD (seropositive for AQP4-IgG) (28), and MOGAD (23) patients, age-matched with or without comorbidities. Participants will undergo an assessment to determine the presence of comorbidity using a predefined list tailored for each organ system. All comorbidities will be listed, including common comorbidities such as cardiovascular diseases and their associated risk factors, as well as autoimmune disorders. For comorbidities with predefined quantitative criteria, screening will be conducted to confirm that patients have advanced beyond the pre-diagnostic stage (e.g., pre-diabetes) to actual diagnosis (e.g., T2DM). In addition to identifying diseases (e.g., ischemic heart disease), we will screen for risk factors such as obesity, hypertension, and hyperlipidemia (29). We will recruit a healthy control group from each region without known chronic conditions or comorbidities matched for age and sex.

#### 2.1.2 Inclusion and exclusion criteria

Eligible participants must possess competence for written informed consent, be ≥18 years of age, and have a diagnosis of MS, NMOSD, or MOGAD with an Expanded Disability Status Scale (EDSS) assessment score from 0 to 6.5 inclusive at the time of recruitment (30, 31).

Patients will be excluded if they are unable to cooperate, have active drug or alcohol abuse, are pregnant, or have an active systemic infection, ON within the preceding 3 months, and steroid treatment in the past 1 month. Dropout criteria will be evaluated during follow-up visits (**Table 1**).

**Table 1:** Inclusion and exclusion criteria.

Inclusion criteria	Exclusion criteria	Dropout criteria
– Competent to give written informed consent – Signed consent—age ≥18 years – Diagnosis of MS (27), NMOSD (28), and MOGAD (23) – Expanded Disability Status Scale (EDSS) assessment score ranging from 0 to 6.5 (inclusive) at baseline	– History of drug or alcohol abuse – Evidence of active systemic infection – Optic neuritis within 3 months prior to the baseline assessment – Any steroid treatment within the preceding month – Pregnancy – Inability to cooperate	– Patient’s withdrawal of consent – Lost to follow-up – Non−compliance with protocol (decision by study board) – Condition hindering study continuation (decision by the study board)

Exclusion criteria are only considered at baseline, while dropout criteria are checked at each follow−up visit.

#### 2.1.3 Medical history and clinical examination

The patients will undergo a clinical examination at baseline visit and on follow-up after 6, 12, 24, and 36 months. Blood sampling, cerebrospinal fluid (CSF) testing, MRI, and OCT will be performed at baseline and annually during the follow-up period. The process is outlined in **Table 2**. Clinical information including age, sex, ethnicity, height, weight, and lifestyle factors such as tobacco and alcohol use, as well as drug abuse history, is recorded. After a combination of interviews and review of past medical records, we will determine disease onset; assess current symptoms; and record histories of relapses or attacks, comorbidities, and comprehensive treatment details, encompassing both pharmacological and non-pharmacological therapies. The neurological evaluation employs the EDSS in accordance with neurostatus definitions (30, 31). The visual system score is determined through best-corrected high-contrast visual acuity testing (15) (**Table 1**).

**Table 2:** Timeline of key assessments and measurements throughout the study period.

Assessment	Detail	Baseline	6 months	12 months	24 months	36 months
**Demographics:**		✓				
Age, sex, weight		✓				
	Body mass index (BMI), waist circumference	✓	✓	✓	✓	✓
Height		✓				
Race/ethnicity	Caucasian/White; Black/African descent; Hispanic/Latino; Asian (including East, South, Southeast, and Central Asian); indigenous/native peoples; Middle Eastern/Arab; mixed/multiple races; other	✓				
Education level	No education; low education (primary/elementary); mid-level education (secondary/high school); vocational/technical training; higher education (undergraduate); advanced education (postgraduate)	✓				
Geographic location	The classification of geographic location is based on the center that prepared the data.	✓				
**Medical history:**	Diagnosis; disease onset; attack/relapse history [number of relapse(s)**]; current symptoms and complaints; vaccination history; previous infections; fertility history	✓	✓	✓	✓	✓
**Comorbid conditions:**	The list of comorbid conditions, along with the corresponding questionnaires and clinical examinations, can be found in **Table 3**. This also includes the date of diagnosis, the severity (when available), the number of comorbid conditions grouped by main categories, and the actual raw numbers.	✓	✓	✓	✓	✓
**Risk factors:**		✓	✓	✓	✓	✓
Smoking	Status: never smoked; former smoker; current smoker Duration: number of years smoking Intensity: average number of cigarettes/day (e.g., 1 pack/day = 20 cigarettes)	✓	✓	✓	✓	✓
Alcohol consumption	Frequency: daily; weekly; monthly; rarely; never Quantity: average number of drinks/sessions	✓	✓	✓	✓	✓
Family medical history Dietary and lifestyle factors	Specific illnesses in first-degree relatives (e.g., cardiovascular disease and diabetes) Level of physical activity	✓		✓	✓	✓
Previous injuries/surgeries	Type of injury/surgery (e.g., fractures, surgeries, and traumatic events)	✓		✓	✓	✓
**Therapies:**	Treatment status; relapse therapy; full drug list	✓	✓	✓	✓	✓
**Neurological examinations**	EDSS***	✓	✓	✓	✓	✓
**Quantitative assessment of motor functions**		✓	✓	✓	✓	✓
Hand grip force	Handheld dynamometer, 3× each side					
Timed tests	T25−FW, 9−HPT					
Visuo−perceptive motion analysis PASS−MS	Short walks, static balance, stand−up and sit, stepping in place, finger tapping, finger−nose test					
**Patient-reported outcomes (PRO)**	Neuro-QOL Fatigue Scale, European Quality of Life–5 Dimensions (EQ-5D) index, EQ-5D visual analog scale (VAS)	✓	✓	✓	✓	✓
**Cognitive assessment**		✓			✓	✓
Interview	Handedness, education	✓			✓	✓
Cognitive function: includes the Montreal Cognitive Assessment (MoCA)	Various cognitive domains such as memory, attention, language, abstraction, delayed recall, orientation, and visuospatial skills	✓			✓	✓
Biospecimens*	Blood samples, cerebrospinal fluid	✓		✓	✓	✓
Vision and the visual system*	Refraction, high− and low−contrast visual acuity, perimetry, VEP, OCT of the macula and optic nerve head, OCT/A (optional: multifocal VEP), NEI-VFQ-25	✓			✓	✓
CNS magnetic resonance imaging^*^	Cerebral: MPRAGE, T2−SPACE, FLAIR, MPM, DWI, rsfMRI Spinal: STIR (whole spine), PSIR (C2 and C3 and C7/T1)	✓			✓	✓

*Further details can be found in the Method section.

**Number of relapse(s) (a new or worsening acute neurologic symptom lasting ≥24 h and not explained by fever or infection).

***Disability assessment with alteration in EDSS score of at least 0.5 from baseline at the clinical visits.

– All are relevant for healthy controls and are only assessed at the baseline visit.

#### 2.1.4 Comorbid conditions

At the start of the study, comorbidity data, including the date of diagnosis, the characteristics, and the associated treatments of the identified comorbidities, will be abstracted from the ‘patients’ medical records (**Table 4**). In cases where medical records are either ambiguous or lack specific details, patients will be interviewed to obtain information on their distinct comorbid conditions. Each comorbidity will be categorized into main categories utilizing global standard codes for primary and secondary diagnoses using the International Statistical Classification of Diseases and Related Health Problem (ICD) system (version 10 or 11) (**Table 3**). For enhanced analytical clarity, patients will be grouped based on whether they have a single comorbid condition or multiple conditions. Special emphasis will be placed on cardiovascular comorbidities for subanalysis. Regular medical assessments at predetermined intervals will enable detection through ICD diagnosis, documentation, and treatment of any new comorbidities.

**Table 3:** Overview of common comorbidities associated with MS, NMOSD, and MOGAD.

Comorbidity categories	Detailed conditions	Severity metrics
Cardiovascular disease	Hypertension, coronary artery disease, heart failure, cardiac arrhythmia, cardiac valvular disease, peripheral vascular disease, smoking, alcohol consumption	ASCVD Risk Calculator: a tool assessing the 10-year and lifetime risks of atherosclerotic cardiovascular disease based on factors like age, cholesterol levels, and blood pressure. Current/former/never smoker
Endocrine, nutritional, and metabolic diseases	Diabetes mellitus type II, thyroid disorders, malnutrition, metabolic disorders, obesity, hyperlipidemia	TSH, BMI Fasting plasma glucose and insulin will be measured to derive the Homeostasis Model Assessment (HOMA) score, indicating glucose tolerance and insulin resistance. For non-diabetic patients, an oral glucose tolerance test (OGTT) will be conducted with samples taken at 0, 30, and 120 min. These measurements will help calculate indices for insulin sensitivity, secretion, and gauge glucose tolerance, including potential impaired glucose tolerance. Additionally, HbA1c will be assessed. Lipid profile (LDL, HDL, TG, cholesterol)
Mental, behavioral, and neurodevelopmental disorders	Insomnia, hypersomnia, sleepwalking [somnambulism], sleep terrors [night terrors], nightmare disorder, circadian rhythm sleep disorders, sleep apnea, narcolepsy and cataplexy, parasomnia, sleep-related movement disorders (RLS), depression, anxiety, bipolar disorder, personality disorders, psychosis, drug abuse, alcohol abuse, fatigue, pain	Beck’s Depression Inventory-II (depression severity), Hamilton Anxiety Rating Scale (anxiety severity), Fatigue Severity Scale (chronic fatigue assessment), FSMC - Fatigue Scale for Motor and Cognitive Functions, Short Form of Brief Pain Inventory (pain evaluation), Pittsburgh Sleep Quality Index and Epworth Sleepiness Scale (sleep quality and propensity), STOP-Bang (sleep apnea risk), Restless Legs Syndrome Rating Scale (RLS severity)
Diseases of the nervous system	Extrapyramidal and movement disorders, Alzheimer’s disease, epilepsy and recurrent seizures, migraine, other headache syndromes, cerebrovascular disease, CNS structural disorders, traumatic brain injury	Hoehn and Yahr staging (Parkinson’s disease progression), seizure frequency (number of epileptic events), Global Assessment of Migraine Severity and frequency of headache per month (migraine and headache assessment)
Diseases of the eye and adnexa	Cataract, glaucoma, visual problems	Visual Functioning Questionnaire-25 and Vision Performance Scale assess visual impairment and its impact on daily activities
Diseases of the respiratory system	Bronchitis, chronic obstructive pulmonary disease, asthma, bronchiectasis, interstitial pulmonary diseases, allergic disease, vascular pulmonary diseases, pleural cavity disease, inflammatory pulmonary disease, non-specific pulmonary diseases	Evaluates lung function, with the GOLD criteria categorizing chronic obstructive pulmonary disease (COPD) severity
Diseases of the digestive system	Irritable bowel syndrome, liver disease, peptic ulcer disease, inflammatory bowel disease, GERD, biliary tract disorders, hemorrhoids and other anal disorders	Patient Assessment of Upper Gastrointestinal Disorder Symptoms (PAGI-SYM) evaluates upper GI symptoms. Alanine transaminase (ALT), aspartate transaminase (AST), alkaline phosphatase (ALP), gamma-glutamyl transferase (GGT), and serum bilirubin are liver function tests
Neoplasms (cancer)	Brain and nervous system cancer, lung cancer, breast cancer, bones and joints cancer, digestive system cancer, endocrine system cancer, eye and orbit cancer, blood cancer, skin cancer, genitourinary system cancer, blood cancer, respiratory system cancer	Stage of cancer: categorizes cancer based on its size, spread, and key characteristics
Diseases of the immune system	Rheumatoid arthritis, other inflammatory arthritis, lupus, celiac disease, Sjogren’s syndrome, sarcoidosis, anti-phospholipid antibody syndrome, ankylosing spondylitis, systemic sclerosis, autoimmune encephalitis, myasthenia gravis, peripheral neuropathies, autoimmune thyroid disease, other non-neurological organ-specific autoimmune diseases	
Diseases of the musculoskeletal system and connective tissue	Gout, arthropathy, arthritis, osteoarthritis, spondylopathy, myositis, osteoporosis, osteopathy, chronic low back pain, fibromyalgia, joint replacement, fracture, spinal stenosis, disc herniation	Nordic Musculoskeletal Questionnaire (NMQ): evaluates musculoskeletal pain
Diseases of the blood and blood-forming organs	Anemias, coagulation defects, blood clots (thrombus) disorder, purpura and other hemorrhagic conditions, hemophilia, sickle cell disease, thalassemia	Hb (hemoglobin level), aPTT, PT, TT (clotting time tests), INR (blood clotting measure), vitamin K, platelets, bleeding time (BT) (platelet function test), clotting factor tests (specific clotting factors), WBC (white blood cell count)
Diseases of the skin	Eczema, psoriasis, pemphigus, alopecia areata, atopic dermatitis, epidermolysis bullosa, Raynaud’s phenomenon, rosacea, hidradenitis suppurativa, scleroderma, ichthyosis, vitiligo, pachyonychia	Dermatological Life Quality Index (impact of skin diseases on life quality), Psoriasis Area Severity Index (psoriasis severity), SCORAD (eczema assessment)
Diseases of the genitourinary system	Glomerular diseases, chronic kidney disease, renal failure, renal tubulo-interstitial diseases, urolithiasis, benign prostatic hyperplasia, disorders of the breast, disorders of the female/male genital tract	Stages of CKD (chronic kidney disease progression), The International Prostate Symptom Score (IPSS) (prostate-related symptoms)
Infections and parasitic diseases	Tuberculosis, malaria, HIV/AIDS, Epstein–Barr virus (EBV), herpesvirus 6, varicella-zoster virus, *Helicobacter pylori*, *Clostridium perfringens*	Antibody level: a measure of specific antibodies in the blood, indicating immune system activity or response to vaccination

**Table 4:** Comorbidity: key terminology and definitions.

Comorbidity (coincidence and concurrent diseases or comorbid conditions)	This general term refers to the presence of one or more additional diseases or disorders occurring concurrently with a primary disease or disorder. Based on **Table 3** categories.
Multimorbidity (polypathology or polymorbidity)	This is the coexistence of two or more chronic diseases or conditions in a patient. The term is often used interchangeably with comorbidity, but while comorbidity refers to the effect of all other diseases an individual patient might have other than the primary disease, multimorbidity refers to the presence of two or more chronic diseases without establishing a primary disease.
Health determinants and indicators	These broader terms encompass a variety of conditions and factors that affect health. For example, in the context of vascular health, this could include both comorbid conditions and risk factors related to vascular diseases.
Comorbid conditions (concurrent disorders and co-existing diseases)	These refer to actual health conditions or diseases an individual has in addition to their primary disease. For instance, in the realm of vascular health, examples could include conditions like heart disease, stroke, and hypertension. These comorbid conditions co-occur with the primary disease and can interact with it in complex ways, potentially impacting treatment and outcomes.
Risk factors (predictors)	These are characteristics or behaviors that increase the likelihood of developing certain diseases. For example, in the case of vascular health, risk factors might include behaviors like smoking, lifestyle factors like lack of exercise or obesity, and genetic predispositions such as family history of vascular disease.

#### 2.1.5 Cognitive assessment

The ‘participant’s level of education and hand preference will be determined through an interview (32). A professional will administer the Montreal Cognitive Assessment (MoCA) in alignment with its official guidelines. MoCA serves as a comprehensive clinical tool designed to evaluate a broad spectrum of cognitive functions related to MS and other conditions (33). This assessment includes cognitive domains such as memory, attention, language, abstraction, delayed recall, orientation, and visuospatial skills. The entire cognitive assessment with MoCA typically takes about 15 min. Additionally, the European Quality of Life–5 Dimensions (EQ-5D) index and the EQ-5D visual analog scale (VAS) (34) will be performed.

#### 2.1.6 Biospecimens

Venous blood and CSF from patients will be collected. CSF will be sampled and stored according to international research standards (35). We will analyze the levels of markers of inflammation and BBB disruption, including matrix metalloproteases (MMPs) (MMP-2, MMP-3, MMP-7, MMP-9, MMP-12, MMP-13), vascular endothelial growth factor A (VEGF-A), and microfibril-associated protein 4 (MFAP4) using enzyme-linked immunosorbent assay (ELISA). Additionally, cytokines and chemokines such as interleukin-1 beta (IL-1beta), IL-6, IL-7, IL-10, IL-17A, tumor necrosis factor alpha (TNF-alpha), C-C chemokine receptor type 2 (CCR2), C-X-C motif chemokine ligand 10 (CXCL10), C-X-C motif chemokine ligand 12 (CXCL12), and C-X-C motif chemokine ligand 13 (CXCL13); type I interferons; neurofilament light chain (NfL); and glial fibrillary acidic protein (GFAP) will be measured as markers of neuronal and astrocyte injury. These biomarkers will be measured on a Quanterix™ Simoa HD-1 Analyzer using the appropriate Simoa assay kits (36).

At baseline and at the 24-month follow-up, peripheral blood mononuclear cells (PBMCs) will be isolated and undergo flow cytometry for quantitative analysis to determine the numbers of CD4, CD8 T lymphocytes, B lymphocytes, dendritic cells, monocytes, monocyte-derived macrophages, FOXP3^+^ regulatory T cells, and FoxA1^+^ Treg and natural killer T cells, as well as mass cytometry for in-depth immune characterization. Additionally, hydrophilic interaction chromatography/mass spectrometry-based proteomics for unbiased screening of plasma/serum will be performed, backed up by targeted highly sensitive assays (Simoa), and RT-qPCR performed centrally. All biospecimens will be collected and analyzed at the University of Southern Denmark.

#### 2.1.7 Patient-reported outcomes

For assessing fatigue, the short-form Neuro-QOL Fatigue Scale (37) will be utilized to measure current levels of fatigue. This scale provides a valuable insight into the impact of fatigue on the quality of life of participants.

#### 2.1.8 Vision and the visual system

Participants will undergo ophthalmological examinations at baseline and at the final visit for the study, which include refraction, high- and low-contrast visual acuity tests, visual fields, and visual evoked potentials (VEPs). Additionally, the National Eye Institute 25-item Visual Function Questionnaire (NEI-VFQ-25) will be utilized to evaluate patients’ visual health-related quality of life (38, 39).

High-resolution OCT images of the retina will be created for all patients and controls (majority of the data for this study will be derived from the Heidelberg Spectralis SD-OCT device as per our retrospective dataset. However, data may be obtained from other OCT devices, including Cirrus HD-OCT, Carl Zeiss; Topcon, Optovue, Canon). The imaging scans for this study will be conducted following the guidelines set forth by the Advised Protocol for OCT Study Terminology and Elements (APOSTEL) 2.0 9-point recommendations (40) and the OSCAR-IB quality criteria (41, 42). We will not consider patients whose documentation is incomplete or who underwent OCT imaging using time-domain devices.

The OCT protocol encompasses various scans such as a peripapillary ring scan, a volume scan of the macula, and a volume scan of the optic nerve head. The measurements taken by the OCT include the peripapillary retinal nerve fiber layer (pRNFL) thickness, ganglion cell-inner plexiform layer (GCIP) thickness, inner nuclear layer (INL) thickness, outer nuclear layer (ONL) thickness, and macular thickness.

Additionally, OCT angiography (OCTA) will be utilized, providing a detailed view of the retinal and choroidal vasculature. Equipment from both Heidelberg and Zeiss will be used to obtain images. Through these images, vascular density, the foveal avascular zone, flow regions, and qualitative vascular irregularities will be assessed. A high standard of image quality will be maintained. Scans meeting an acceptable signal strength index will be selected for analysis centrally, and those compromised by artifacts or other quality issues will be carefully excluded (1). All OCT data will be collected at Charite University, Berlin, Germany.

#### 2.1.9 CNS MRI

In the comparative analysis of MRI findings for the index disease, both with and without comorbidity, our initial primary focus centers on vascular comorbid conditions. MRI scans will be conducted using a 3T system compatible with Siemens, Philips, and GE scanners. The core structural protocol includes the brain, orbit, and spinal cord.

##### 2.1.9.1 Brain MRI

The core structural protocol will comprise 3D fluid-attenuated inversion recovery (FLAIR), T2 sampling enhanced with specialized contrasts (SPACE), susceptibility-weighted imaging (SWI), and T1 magnetization prepared-rapid gradient echo (MPRAGE) sequences (optimized for 1-mm isotropic pixels). Post-contrast T1 imaging will be performed in cases of acute relapse (~45 min) (43, 44).

Additionally, the research protocol for brain MRI for selective patients includes a specialized subgroup measurement that involves multiparametric mapping (MPM) for detailed tissue characterization, extending the scan duration by approximately 20 min (45, 46). Procedures such as diffusion-weighted imaging (DWI) (47), resting-state functional MRI (RS-fMRI), and advanced sequences like susceptibility-weighted imaging (SWI) and T2*-weighted imaging will be performed, with durations estimated at 3–5 min each (a total of ~30 min) (48).

##### 2.1.9.2 Spinal cord

The protocol includes a 3D T2 SPACE sequence, a 3D T1 MPRAGE sequence for the cervical cord, and a 3D T2 sequence for the thoracic cord (~12 min).

##### 2.1.9.3 Optic nerve

The MRI protocol will feature a high-resolution, 3D T2-weighted orbit sequence focused on the optic nerves with a slice thickness of 2 mm or less. Fat suppression techniques will be employed to enhance contrast and reduce artifacts, and a post-contrast T1-weighted sequence may also be used to assess inflammatory or demyelinating changes in the optic nerve (~10–15 min) (49–52). All MRI data will be evaluated at the University of Oxford, UK.

#### 2.1.10 Outcome measures and assessment timeline

**Figure 1:**
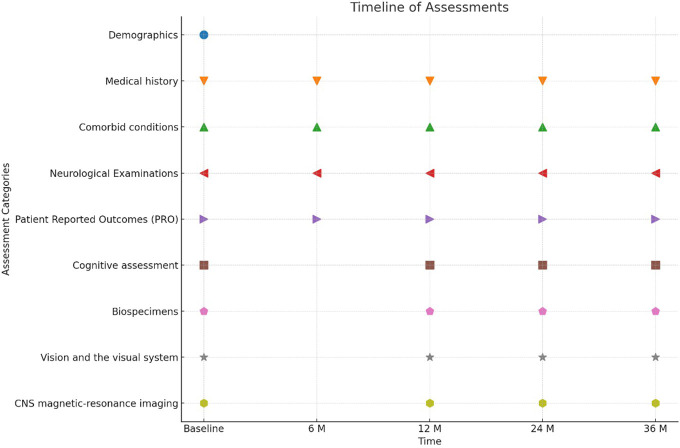
Timeline of patient assessments and interventions in a clinical study spanning 36 months.

In this study, a comprehensive set of outcome parameters, as detailed above, will be monitored and analyzed at each clinical visit to the participating centers (**Table 2**). Assessments are scheduled to be conducted at the initiation of the study (baseline) and subsequently at 6, 12, 24, and 36 months. This scheduling allows for the tracking of changes over time (**Table 2**, **Figure 1**). As an interim analysis specifically oriented at regional differences, we will conduct subgroup analyses to explore cross-sectional region-specific effects.

#### 2.1.11 Assessments and endpoints

In MS, the evaluation of disease progression is refined through advanced measures, with a primary focus on no evidence of disease activity (NEDA-4) (40, 53, 54). NEDA-4 assessment in our study includes four criteria: no clinical relapses; no new/enlarged T2-weighted MRI lesions; no disability progression over 6 months, assessed by the EDSS score; and a mean annual brain volume loss rate under 0.4%.

For NMOSD and MOGAD, the primary endpoint is relapse frequency [annualized relapse rate (ARR)] and time to relapse. Secondary endpoints are clinically important change from baseline in Hauser Ambulation Index (HAI) score (55), changes from baseline in EQ-5D index and EQ-5D visual analog scale (VAS), disability worsening from baseline in EDSS score (30) and change from baseline in low-contrast visual acuity binocular score (by low-contrast Landolt C broken ring chart), and cumulative total number of active MRI lesions (new gadolinium-enhancing lesions, or new or enlarging T2 lesions, measured across the optic nerve, brain, brainstem, and spinal cord).

The number of IDD-related hospital admissions will be recorded. Patient-reported outcomes also provide further insights into the patient’s experience of the disease in this study (56). Exploratory outcomes include OCT parameters and clinically relevant biomarkers like NfL levels and GFAP for NMOSD (57, 58).

#### 2.1.12 Data management and quality control

Data associated with the study will be stored within the REDCap database system (59). Monthly data monitoring will be conducted using a REDCap query function for completeness and accuracy by the research team to ensure that any errors or inconsistencies are identified and addressed in a timely manner.

### 2.2 Streamlined dataset protocol for multicenter coordination

To achieve consistent data collection across various centers, it is crucial to establish a standardized minimal dataset. This standardized approach is key to facilitating data sharing and analysis, ensuring uniformity and reliability in study outcomes across diverse facilities. It is planned that the specifics of the data subsets for each center will be thoroughly discussed with all participating centers. A primary milestone for the multicentric part of this project is the initiation of cross-sectional data collection.

### 2.3 Statistical analysis

The data will be analyzed in two stages: first cross-sectionally and then longitudinally. The cross-sectional analysis will focus on examining the relationships among the variables at a single point in time, while the longitudinal analysis will explore changes in these relationships over time.

In the initial stage of analysis, the data will be evaluated cross-sectionally. This will encompass descriptive statistics to summarize central aspects like means, medians for continuous variables, and frequencies for categorical ones. Additionally, correlation analyses will gauge the linear relationships between pairs of variables, while regression analysis will investigate the predictive influence of independent variables on a designated dependent variable. In the subsequent phase, the focus will be on the evolution and patterns in relationships over time. Repeated measures ANOVA will be employed to compare means across different times and identify any significant changes. Growth curve modeling will be done to understand the trajectory of variables over time and to determine if there are individual differences in the change trajectories. Furthermore, to accommodate instances of missing data or irregular time intervals, mixed-effects models will be utilized, considering both fixed and random influences.

To determine the sample size, the study’s primary objectives and expected effect sizes will be considered by using R statistical software. *A-priori* power analyses will be utilized to establish the needed sample size with an optimal power, usually set at 0.80, for a typical significance level of 0.05. Practical significance of the observed relationships or differences will be assessed.

Association analyses of outcomes with comorbidity and age (including comorbidity–age interactions) as well as relations between the different outcomes will be a major focus. Predictive modeling of disease progression will be the content of a follow-up analysis beyond this project’s period, where we intend to use both disease biomarkers and the related risk factor markers for vascular comorbidities as predictors together with techniques from machine learning and algorithm-based data analyses.

The study has a confounder bias, as subjects with comorbidity will receive treatment as the standard of care at different time points. Comorbidity treatment initiated between the baseline and follow-up will be analyzed as potential confounders. The influence of confounders will be minimized by advanced biostatistical techniques, including confounder adjustment.

### 2.4 Discussion

Comorbidity refers to the total burden of any additional disease entity other than the specific index disease and may result from predisposing genetic or environmental factors or from treatment for IDDs. Comorbidity is prevalent among people with IDDs of the CNS, consistent with the rising prevalence of multimorbidity, estimated to affect approximately 40% of the European population. The prevalence of comorbidity not only signifies elevated mortality rates but also correlates with heightened healthcare resource utilization (17). Comorbidities may have decisive importance in terms of diagnosis, prognosis, and treatment effectiveness and safety. The scarcity of established modifiable risk factors for the burden of multimorbidity indicates that new knowledge is required to develop effective prevention and treatment strategies.

The COMMIT research program focuses on the three best-defined IDDs: MS, NMOSD, and MOGAD. NMOSD and MOGAD are antibody-associated diseases of the CNS. In NMOSD, an autoantibody targets the astrocyte water channel AQP4 and defines NMOSD as a primary astrocytopathy in AQP4-IgG-positive cases (60, 61). In MOGAD, an autoantibody targets myelin antigens, i.e., MOG, which is produced by oligodendrocytes and thus is involved in demyelination (62). MS is thought to be an autoimmune disease and to be a mainly myelin-directed disease although causative specificities have not been identified, and considerable overlap may occur between MS and MOGAD. The goal of the present study is to assess whether comorbidity has an impact on the levels of proinflammatory and neurodegenerative mediators and in practical terms whether they influence OCT and MRI parameters and is associated with clinical outcome.

In the COMMIT study, the patients with IDDs and comorbidity are the primary target group. This project advances the management of IDD patients with chronic comorbidities and may contribute to the pathogenesis of diseases. Identification of reliable biomarkers will allow evaluation of the impact of comorbidity on IDDs and enable early diagnosis and new therapeutic strategies. Our prospective multicenter study, which will be conducted over a 3-year follow-up period, aims to compile a detailed and systematic database for patients who exhibit various comorbid conditions.

A strength of the study is the multicenter design, with longitudinal, consecutive follow-up with sample collection and a uniform protocol. Given the exhaustive nature of the study protocol, it can be particularly challenging for individuals with disabilities. We will introduce a streamlined, standardized assessment to ease follow-ups. With an adequate sample size, our objective is to curtail the effects of confounding.

## 3 Conclusion

In conclusion, the intricate relationship between IDD and other chronic conditions represents an area for further research. The COMMIT study will address if the clinical and biochemical status of patients with MS, NMOSD, and MOGAD is affected by comorbid conditions. In addition, the study is cross-disciplinary, contributing to the characterization of disease mechanisms. The study will identify clinically relevant biomarkers, as well as address survival and quality of life. Recognizing the frequency and patterns of comorbidities and risk factors can be instrumental in developing preventive strategies. The results of the study are anticipated to have a strong and immediate impact on the perception and treatment of IDDs due to improvement in diagnostic and monitoring biomarkers.

## Data availability statement

The original contributions presented in the study are included in the article/supplementary material. Further inquiries can be directed to the corresponding author.

## Ethics statement

The Research Ethical Committees for the Region of Southern Denmark approved the study protocol. Approval by the local ethical institutional review boards from all centers for this multicenter study was obtained following informed consent from the patients. The studies were conducted in accordance with the local legislation and institutional requirements. The participants provided their written informed consent to participate in this study.

## Author contributions

SS: Visualization, Formal analysis, Methodology, Investigation, Writing – review & editing, Writing – original draft. RA: Writing – review & editing, Writing – original draft. PB: Writing – review & editing, Writing – original draft. MB: Investigation, Writing – review & editing, Writing – original draft. BD: Formal analysis, Methodology, Writing – review & editing, Writing – original draft. SR: Methodology, Writing – review & editing, Writing – original draft. ML: Supervision, Resources, Investigation, Writing – review & editing, Writing – original draft. AP: Supervision, Methodology, Investigation, Writing – review & editing, Writing – original draft. JP: Validation, Supervision, Methodology, Investigation, Writing – review & editing, Writing – original draft. EF: Validation, Supervision, Methodology, Investigation, Writing – review & editing, Writing – original draft. SMa: Supervision, Methodology, Investigation, Writing – review & editing, Writing – original draft. STS: Writing – review & editing, Writing – original draft. AF: Validation, Supervision, Methodology, Investigation, Writing – review & editing, Writing – original draft. IL: Methodology, Investigation, Writing – review & editing, Writing – original draft. SMe: Methodology, Writing – review & editing, Writing – original draft. RG: Methodology, Investigation, Writing – review & editing, Writing – original draft. SA: Methodology, Investigation, Writing – review & editing, Writing – original draft. HS-K: Methodology, Investigation, Writing – review & editing, Writing – original draft. FO: Supervision, Methodology, Investigation, Writing – review & editing, Writing – original draft. VS: Supervision, Investigation, Writing – review & editing, Writing – original draft. MS: Supervision, Investigation, Writing – review & editing, Writing – original draft. HK: Validation, Supervision, Methodology, Investigation, Writing – review & editing, Writing – original draft. JB: Validation, Supervision, Methodology, Writing – review & editing, Writing – original draft. CB: Methodology, Investigation, Writing – review & editing, Writing – original draft. HZ: Supervision, Methodology, Investigation, Writing – review & editing, Writing – original draft. BW: Validation, Supervision, Investigation, Writing – review & editing, Writing – original draft. FP: Validation, Supervision, Methodology, Investigation, Writing – review & editing, Writing – original draft. NA: Validation, Supervision, Project administration, Methodology, Investigation, Funding acquisition, Conceptualization, Writing – review & editing, Writing – original draft.

## Glossary

AT2R: angiotensin II type 2 receptor
BBB: blood–brain barrier
CCR2: C-C motif chemokine receptor 2
CD: cluster of differentiation
CNS: central nervous system
CSF: cerebrospinal fluid
CXCL: C-X-C motif chemokine ligand
DMT: disease-modifying therapy
EDSS: Expanded Disability Status Scale
ELISA: enzyme-linked immunosorbent assay
FOXP3: forkhead box P3
FoxA1^+^ Treg: forkhead box A1-positive regulatory T cells
GCIP: ganglion cell-inner plexiform layer
GFAP: glial fibrillary acidic protein
IDDs: inflammatory demyelinating diseases
IL: interleukin
INL: inner nuclear layer
MFAP4: microfibril-associated protein 4
MOG: myelin oligodendrocyte glycoprotein
MOGAD: MOG-associated disease
MRI: magnetic resonance imaging
MMPs: matrix metalloproteases
MS: multiple sclerosis
NEI-VFQ25: the National Eye Institute 25-item Visual Function Questionnaire
NfL: neurofilament light
NMDAR EN: N-methyl-D-aspartate receptor encephalitis
NMOSD: neuromyelitis optica spectrum disorder
OCT: optical coherence tomography
ON: optic neuritis
ONL: outer nuclear layer
OPC: oligodendrocyte precursor cell
OL(s): oligodendrocytes
PBMCs: peripheral blood mononuclear cells
pRNFL: peripapillary retinal nerve fiber layer
PPMS: primary progressive MS
QoL: quality of life
REDCap: Research Electronic Data Capture
RRMS: relapsing–remitting MS
RT-qPCR: real-time quantitative polymerase chain reaction
SD-OCT: spectral domain optical coherence tomography
SPMS: secondary progressive MS
T2DM: type 2 diabetes mellitus
TNF-alpha: tumor necrosis factor alpha
TREM2: triggering receptor expressed on myeloid cells 2
pTau-181: phosphorylated tau at position 181
VEGF-A: vascular endothelial growth factor-A.
